# Elevated CXCL-8 expression in bronchoalveolar lavage correlates with disease severity in patients with acute respiratory distress syndrome resulting from tuberculosis

**DOI:** 10.1186/1476-9255-11-21

**Published:** 2014-08-05

**Authors:** Seyed Mohamad Reza Hashemian, Esmaeil Mortaz, Payam Tabarsi, Hamidreza Jamaati, Zohreh Maghsoomi, Adnan Khosravi, Johan Garssen, Mohamad Reza Masjedi, Ali Akbar Velayati, Gert Folkerts, Peter J Barnes, Ian M Adcock

**Affiliations:** 1 Chronic Respiratory Diseases Research Center, National Research Institute of Tuberculosis and Lung Diseases, Shahid Beheshti University of Medical Sciences, Tehran, Iran; 2 Division of Pharmacology and Pathophysiology Utrecht Institute for Pharmaceutical Sciences, Faculty of Sciences, Utrecht University, Utrecht, The Netherlands; 3 Department of Infectious Diseases, Mycobacteriology Research Center, National Research Institute of Tuberculosis and Lung Diseases (NRITLD), Masih Daneshvari Hospital, Shahid Beheshti University of Medical Sciences, Tehran, Iran; 4 Danone Research Centre for Specialised Nutrition, Wageningen, The Netherlands; 5 Airways Disease Section, National Heart and Lung Institute, Imperial College London, London, UK

**Keywords:** ARDS, TB, CXCL8 and neutrophils

## Abstract

**Background:**

Tuberculosis (TB) is a rare but known cause of acute respiratory distress syndrome (ARDS). The role of inflammatory cytokines in the progression of ARDS in TB patients is unknown.

**Objectives:**

In this study we investigated the possible link between the levels of inflammatory cytokines in bronchoalveolar lavage (BAL) in patients with TB or ARDS alone or in patients with TB-induced ARDS (ARDS + TB).

**Methods:**

90 patients were studied: 30 with TB alone, 30 with ARDS alone and 30 with ARDS + TB. BAL was collected by fiberoptic bronchoscopy and the concentrations of interleukin(IL)-6, CXCL8, TNF-α and IL-1β and the amounts of total protein were measured by ELISA and bicinchoninic acid assay (BCA) methods respectively. The correlation between disease severity measured by Murray scores, SOFA and APACHE II analysis and BAL mediators and cells was also determined.

**Results:**

CXCL8 levels in BAL were significantly higher in the ARDS + TB group compared to TB and ARDS alone groups. Disease severity in the ARDS + TB group as determined by Murray score correlated with BAL CXCL8 and neutrophils but not with IL-6, IL-1β and TNF-α concentrations. In addition, CXCL8 levels and neutrophils were increased in non-miliary TB versus miliary TB. This difference in CXCL8 was lost in the presence of ARDS.

**Conclusions:**

BAL CXCL8 levels were significantly higher in patients with ARDS induced by TB and could suggest an important role of CXCL8 in the pathogenesis of this form of ARDS. This further suggests that CXCL8 inhibitors or blockers may be useful to control the onset and/or development of these combined diseases.

## Introduction

Acute respiratory distress syndrome (ARDS) is a common disorder in the intensive care unit (ICU) and is associated with high mortality and morbidity [1]. The common causes include sepsis, pneumonia and aspiration. Tuberculosis (TB) remains a major public health problem in most of the developing world and the emerging epidemic of acquired immunodeficiency syndrome has resulted in a resurgence of TB throughout the world [2]. Tuberculosis is a disease of protean manifestations with the lungs being the most commonly involved site. Untreated pulmonary TB carries a mortality of 50% with the most common cause of death being extensive fibrocavitary disease and respiratory failure [3]. Miliary tuberculosis is a life-threatening disease caused by the haematogenous spread of Mycobacterium tuberculosis. It also features as an unusual cause of ARDS. ARDS is independently associated with mortality in TB patients in ICU [4] and is inversely associated with the treatment of underlying TB in those patients [5]. Factors contributing to the high mortality rate in ICU include consolidation on chest radiographs, multiple organ failure [6], high Acute Physiology and Chronic Health Evaluation (APACHE) II scores and sepsis [7]. Although there have been several case reports describing the association of ARDS and pulmonary TB [7-10], TB is rarely recognized as a cause of ARDS. In fact, TB does not feature in most recent reviews on ARDS [11,12]. Although it is known that IFN-γ plays an important role in the pathogenesis of TB infection, a complex network of other cytokines, such as tumour necrosis factor alpha (TNF-α) [13], CXCL8 [14-16], and IL-1β [17] are also thought to be important in the pathogenesis of disease.

ARDS is a devastating inflammatory disease of the lung which is characterised by the sudden onset of increased pulmonary vascular permeability, pulmonary oedema and respiratory failure. Activated neutrophils play a major role in mediating the microvascular damage and also contribute to lung tissue injury. Polymorphonuclear (PMN) cells infiltrate the lung in significant numbers and their persistence in the lungs is an important determinant of poor survival [18,19].

CXCL8 has been identified as one of the most significant chemotactic factors for PMN in the blood and bronchoalveolar lavage (BAL) fluid in patients with ARDS [20,21]. In addition, CXCL-8 has been shown is necessary for granuloma formation in the rabbit model of TB [22].

CXCL8 in TB-infected tissue may also have effects in addition to its actions on chemotaxis being both anti-apoptotic [23], and pro-angiogenic [24]. CXCL8 has also been reported to affect intracellular pathogen survival, promoting the survival of *Chlamydia pneumoniae* within neutrophils [23], but increasing the killing of neutrophil phagocytosed avirulent mycobacteria [25]. However, the neutrophil is not the target cell of *M tuberculosis* and there are no data suggesting that CXCL8 affects the survival of *M tuberculosis*. CXCL8 secretion in TB-infected tissues is largely attributed to leukocytes. Monocytes secrete CXCL8 and cytokines in response to *M tuberculosis*[26-28]. CXCL-8 is also released by diverse BAL cells including monocytes and macrophages and other cell types, including fibroblasts [29], and the pulmonary epithelium [30]. Interestingly, Yang et al. reported that the absence of CXCL8 from TB pleural effusions [31].

In a series of studies, Donnelly et al. studied a heterogeneous group of ICU-patients with a predisposition for ARDS due to trauma, pancreatitis or bowel perforation [32]. Generally, ICU patients with a BAL CXCL-8 concentration higher than 200 pg/ml developed ARDS 6–72 h following admission [32], whereas, only one patient with a BAL CXCL-8 concentration lower than 200 pg/ml developed ARDS. In addition to CXCL-8, pulmonary concentrations of IL-6, TNF-α and IL-1β were also increased either during the development of, or during the early phase of, ARDS [33-37].

Although TB is a treatable illness, there is a persistently high mortality (69–80%) in patients with severe pulmonary TB and acute respiratory failure [38,39], probably as a result of TB not being recognised as a cause of ARDS and acute respiratory failure [40].

Thus, in current study we aimed to investigate the levels of BAL CXCL8 and related cytokines and cells in TB, ARDS or in the combined condition to finding the possible links with disease severity. We report that BAL levels of CXCL-8 are raised in patients with ARDS + TB and that this correlates with the severity of ARDS.

## Materials and methods

### TB and ARDS patients

The study was reviewed and approved by the Masih Daneshvari Hospital Ethics Committee and was conducted in accordance with the 2000 Declaration of Helsinki. Informed consent was obtained from all patients or their accompanying adult.

Inclusion criteria included patients in ICU with ARDS, TB or TB accompanied with ARDS [5,41]. 20 disease controls with a negative purified protein derivative (PPD) test were also included in the study. Patients were included if they were at risk for ARDS and meeting predefined criteria for either sepsis or severe trauma. Patients with ARDS met the following criteria according to the Berlin classification of ARDS [41].

The percentage of ARDS patient on the Berlin category according to PaO2/FiO2 ratio was 10% mild form, 55% moderate and 35% with severe form. The Murray score was used to quantify the clinical severity of ARDS. Survival was defined as discharge from hospital.

90 subjects with suspected active pulmonary TB disease, who had negative results in acid fast bacilli (AFB) smears from sputa, and who had consequently undergone BAL procedure for diagnostic purposes were enrolled. Ventilatory strategies were similar in all patients. Patients with a confirmed diagnosis of TB were assigned to the TB group. If the TB patient admitted to ICU contracted ARDS after admission the patient was assigned to the ARDS + TB group. Exclusion criteria for the study were patients less than 15 years of age or more than 80 years of age and patients with known underlying pulmonary disorders other than TB. Those who died within the first 24 h were also excluded.

### Murray score calculations

The Murray score was calculated as previously described [42]. The Murray scoring system includes 4 criteria for the development of ALI/ARDS: a “scoring” of hypoxaemia, a “scoring” of respiratory system compliance, chest radiographic findings, and level of Positive End Expiratory Pressure (PEEP). Each criterion receives a score from 0 to 4 according to the severity of the condition. The final score is obtained by dividing the collective score by the number of components used. A score of zero indicates no lung injury, a score of 1 to 2.5 indicates mild to moderate lung injury. Generally a final score of more than 2.5 indicates the presence of ARDS. In this particular study a Murray score of ≥3 was the minimum entry criteria for the definition of ARDS.

### Evaluation the APACHE score of study patients

The severity of disease was also assessed based on the Acute Physiology and Chronic Health Evaluation (APACHE) score [43-45]. The effects of age and chronic health status are incorporated directly into the model, weighted according to their relative impact, to give a single score with a maximum of 71. The worst value recorded during the first 24 hours of a patient’s admission to the ICU was used for each physiological variable. The principal diagnosis leading to ICU admission was added as a category weight. The severity of organ dysfunction was also assessed using the Logistic Organ Dysfunction System which was scored based on the sequential organ failure assessment (SOFA) calculation where organ failure was defined by an SOFA score of 3 or more [31].

### BAL fluid collection and differential cell counts

BAL fluid was collected by fibrotic bronchoscopy from all the groups (TB, ARDS, TB + ARDS and patients who underwent a BAL for another reason) with ARDS within 24 h of diagnosis being established. All the patients were mechanically ventilated with 100% FiO_2_. Six aliquots (20 mL each) of sterile normal saline were instilled and the fluid was aspirated immediately after each instillation. The first retrieved BAL sample, reflecting a bronchial sample, was discarded and the remaining BAL was pooled in ice-cold tubes and stored at -20°C. The BAL was centrifuged at 200xg for 10 min at 4°C to obtain the supernatant. Cell-free BAL fluid specimens were aliquoted and stored at -80°C until analysis. The samples were frozen approximately 30 min after the bronchoscopy procedure. All assays were performed at the same time on defrosted samples. The BAL fluid was centrifuged (150x*g*, 5 min, 4°C), and the cell-free supernatant was divided into aliquots and frozen at -80°C. BAL fluid was filtered through sterile gauze to exclude mucus plugs and was then centrifuged to obtain a cell pellet. The cell pellet was washed once in 50 ml of Ca^2+^/ Mg^2+^ free Hanks' balanced salt solution (HBSS). The cells were counted on a hemocytometer slide using a Kimura counterstain and viability assessed by trypan blue exclusion. Cytospins were performed, using 10 [4] cells per slide, and stained with May-Grunwald-Giemsa in order to obtain differential cell counts.

### Cytokines ELISA assays

BAL levels of IL-6, CXCL-8 (BD, USA), IL-1β (Invitrogen, USA), and TNF-α (bioscience, USA) were measured by ELISA kits according the manufactures’ instructions.

### Total protein assays

Total protein determinations were performed on BAL samples using the Micro BCA (bicinchoninic acid) protein assay reagent kit (Pierce Biotechnology, USA) according to the manufacturer’s instructions. In brief, each sample was diluted 1:20 with the Working Reagent, incubated at 37°C for 30 minutes before reading in a spectrophotometer at 562 nm. Values were compared to that for a freshly prepared protein standard curve.

### Statistical analysis

All multiple comparison tests were two-tailed. Direct comparisons between two treatment groups were performed with the unpaired Student t-test or the nonparametric Mann–Whitney test when the data sets were not normally distributed. Pearson’s correlation of coefficient was used to analyze correlations between the inflammatory mediators and the various parameters measured. A p value of 0.05 or less was considered significant. All statistical analyses were performed with GraphPad Prism6.

## Results

### Severity of ARDS in patient groups

A total of 90 patients (32 females/58 male, mean age 38.8 yrs) admitted to ICU were included in the study. Thirty patients had ARDS due to other complication such as sepsis, pneumonia and autoimmune vasculitis, 30 had TB only and were admitted to ICU due to pneumonia and pneumothorax and 30 developed ARDS on the background of TB. Eleven of these patients had miliary TB which progressed to ARDS in ICU and 19 had TB with ARDS. 20 disease controls (patients who underwent a BAL for another reason) with a negative purified protein derivative (PPD) test were also included in the study as a control group. The disease control ICU patients had a variety of causes with 8/20 having an infectious aetiology versus 12/20 having a non-infectious aetiology. The mean APACHE II score, SOFA score and Simplified Acute Physiology Score (SAPS II) at the time of admission was estimated and recorded for each patient (see Table 1). 58 (64.4%) patients required mechanical ventilation. Patients with either TB or ARDS alone were matched and selected as control groups. The ICU mortality rate was 32.4%. The mean survival time of the patients with ARDS (with or without TB) who died was 36.3 days (range 1–69), with 45% of the patients dying within the first 30 days.

**Table 1:** Control subjects are age-matched with a negative PPD (purified protein derivative) test

	**Controls**	**ARDS**	**TB**	**ARDS + TB**	**P-value**
**Age**	42 ± 4.3	40 ± 5.8	42 ± 6.2	37 ± 7.5	P > 0.05
**Male/Female**	13/7	21/9	18/12	17/13	P > 0.05
**Weight (kg)**	70/75	69 ± 17.6	70 ± 14.8	74 ± 12.5	P > 0.56
**Height (m)**	163 ± 5	168 ± 12.5	170 ± 9.6	172 ± 8.5	P > 0.075
**BMI**	25 ± 3.3	24.1 ± 5.2	25.7 ± 7.5	21.5 ± 5.8	P > 0.45
**BAL volume**	25 ± 4.3%	23.5 ± 8.2	26.7 ± 7.3	28.1 ± 9.9	P > 0.05
**APACHE II score**	0	22.7 ± 3.9	18.5 ± 6.2	17.41 ± 5.6	**P < 0.05**
**SOFA score**	0	13.6 ± 2.7	12.32 ± 3.1	14.25 ± 2.5	P > 0.05
**SAPS score**	0	30.3 ± 7.2	32.5 ± 5.5	41.25 ± 12.05	P > 0.05

*Abbreviations*:

*APACHE II* Acute Physiology and Chronic Health Evaluation.

*ARDS* Acute Respiratory Distress Syndrome.

*BAL* bronchoalveolar lavage.

*BMI* body mass index.

*SOFA* Sequential Organ Failure Assessment.

*SAPS* Simplified Acute Physiology Score.

*TB* Tuberculosis.

The age and other demographic factors and mean (±SD) of APACHE II, SOFA, and SAPS II scores were compared between the three groups (Table 1). There was no significant difference in any of the measures on admission except for patients in the ARDS alone group who had a significantly higher APACHE II score than subjects in the other two disease groups (Table 1).

### Patients with TB and ARDS have higher levels of BAL CXCL-8

**Figure 1:**
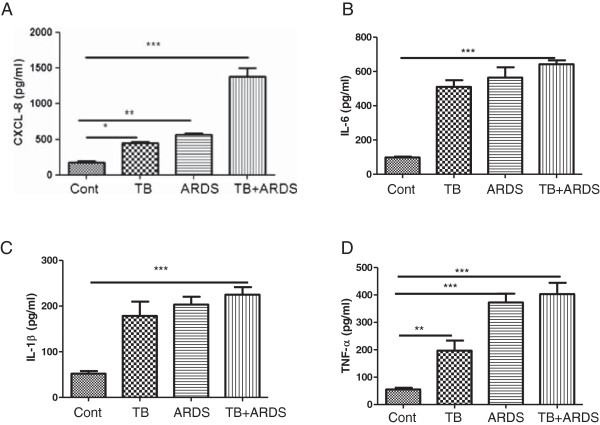
**Cytokines levels in bronchoalveolar lavage (BAL) of patients with tuberculosis (TB), acute respiratory distress syndrome (ARDS) and the combination of both TB and ARDS.** BAL CXCL8 **(A)**, IL-6 **(B)**, IL-1β **(C)** and TNFα **(D)** levels in patients with TB, ARDS and in combined patients were measured by ELISA as described in material and methods. Control subjects are age-matched with a negative PPD (purified protein derivative) test. Data are presented as mean ± SEM (n = 30 in each group except for controls where n = 20). *p ≤ 0.05, **p ≤ 0.01 and ***p ≤ 0.01 compared with control.

CXCL-8 concentrations in BAL were significantly elevated in the TB alone and ARDS alone groups compared to disease controls (patients who underwent a BAL for another reason) and further increased in the ARDS + TB group (Figure 1A). The concentrations of IL-6 were similar in all groups and were significantly elevated compared to the control group (Figure 1B). Similar results were observed for the BAL concentrations of IL-1β (Figure 1C). The concentration of BAL TNF-α was significantly greater than control levels in the TB group and was further increased in the ARDS alone and in the ARDS + TB group (Figure 1D). There was no significant difference between the levels of TNF-α in the TB alone group and the other disease groups.

### Patients with TB and ARDS have higher levels of BAL total proteins

**Figure 2:**
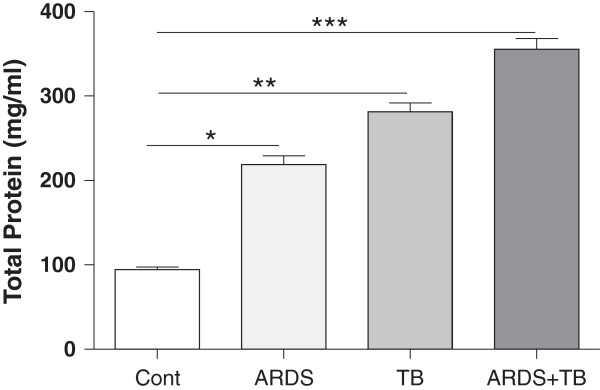
**The total protein content of bronchoalveolar lavage (BAL) of patients with tuberculosis (TB), acute respiratory distress syndrome (ARDS) and the combination of both TB and ARDS.** After obtaining the BAL fluids from TB, ARDS and combination, the levels of total protein was determined as described at materials and methods. Data are presented as mean ± SEM (n = 30 in each group except for controls where n = 20). *p ≤ 0.05, **p ≤ 0.01 and ***p ≤ 0.01 compared with control.

In addition, BAL total protein levels were higher than those in control subjects in all three patient groups (Figure 2). The levels seen in the ARDS + TB group were the highest and these were significantly higher than those in the ARDS alone group (P < 0.05).

### Patients with TB and ARDS has higher levels of neutrophils in BAL

**Figure 3:**
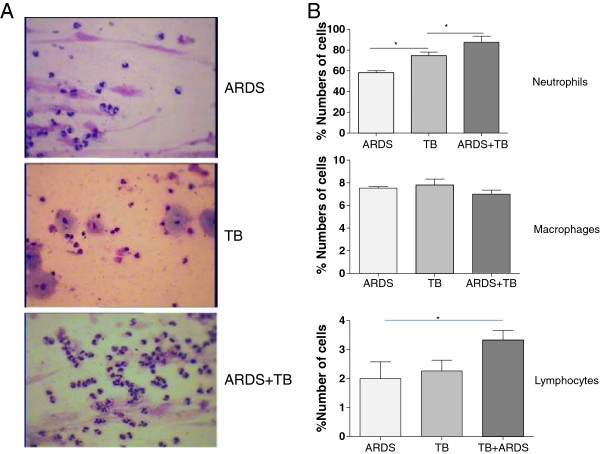
**Differential cell counts in bronchoalveolar lavage (BAL) of patients with tuberculosis (TB), acute respiratory distress syndrome (ARDS) and the combination of both TB and ARDS.** Cytospins of BAL cells were distinguished by diff Quick staining and by morphology (Hemacolor, original magnifi cation x 1,000) **(A)**. Total cells were counted and the percentage of each cell type was calculated when the counted total cell numbers were more than 200 and represented as neutrophils, macrophages and lymphocytes **(B)**. The data among groups were analyzed using one-way analysis of variance, and the differences between two groups were tested using an unpaired *t* test; *P* ≤ .05 was considered significant. Data are presented as mean ± SEM (n = 30 in each group). *p ≤ 0.05 between groups.

Differential cell counts were performed on cytospins obtained from BAL fluid. In BAL, the most abundant cells observed in the ARDS, TB and TB + ARDS groups were neutrophils (Figure 3A) which comprised 67.8 ± 5.8%, 78.2 ± 3.0 and 91 ± 4.4% of the total cells in each sample, respectively (Figure 3B). The numbers of BAL neutrophils were significantly greater between TB alone and ARDS alone groups (P < 0.05) and between the TB alone and ARDS + TB groups.

**Figure 4:**
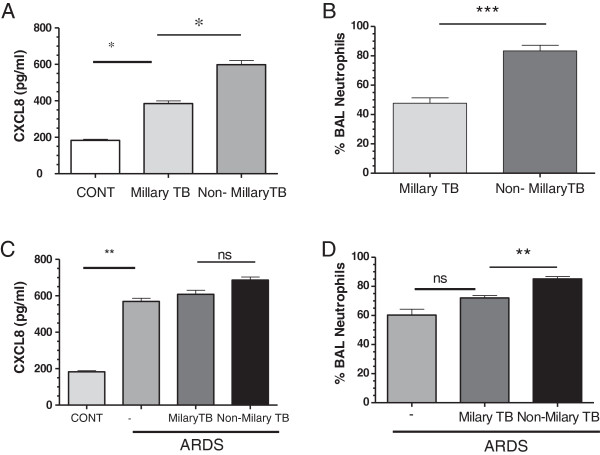
**CXCL8 levels and neutrophil numbers in bronchoalveolar lavage (BAL) of patients with miliary and non-miliary tuberculosis (TB).** BAL CXCL8 levels **(A)** and the percentage BAL neutrophils **(B)** in patients with miliary (n=11) and non-miliary TB (n=19) were measured by ELISA as described in the materials and methods. The effect of the presence of acute respiratory distress syndrome (ARDS) on CXCL8 **(C)** and BAL neutrophils **(D)** was also assessed. Control (CONT) subjects are age-matched with a negative PPD (purified protein derivative) test. Data are presented as mean±SEM. *p≤0.05, **p≤0.01 and ***p≤0.01.

Macrophages were much less numerous than neutrophils in all these samples (7.8 ± 3%, 7.8% ± 4.1% and 5.9 ± 4% in BAL, respectively), and lymphocytes comprised only about 2-3% of the total cells found in BAL. The remaining cells were epithelial cells (data not shown). There were no significant differences between the groups for any of these cell types.When we analysed the data according to whether the subjects had milary or non-milary disease there was a significant increase in BAL CXCL-8 (385.1 ± 14.8 vs 598 ± 22.6 pg/ml, p < 0.05) (Figure 4A) and neutrophilia (46.7 ± 4.1 vs 68.9 ± 2.5%, p < 0.001) (Figure 4B) in the patients with non-milary TB compared to those with milary TB. The significant increase in BAL CXCL-8 (608.6 ± 21.8 vs 686.5 ± 16.4 pg/ml, p = ns) (Figure 4C) was not observed in those patients with ARDS. In contrast, the difference in BAL neutrophils remained significant (72.0 ± 1.7 vs 85.1 ± 1.6%, p < 0.01) (Figure 4D).

### Correlation of TB and ARDS with mortality of patients

**Figure 5:**
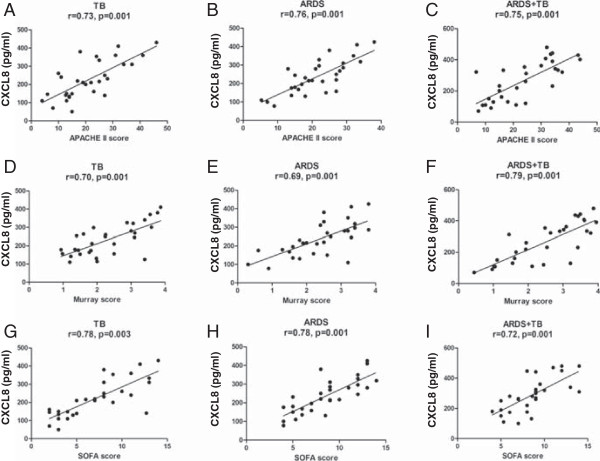
**Correlations between CXCL8 levels in bronchoalveolar lavage (BAL) and patient mortality.** BAL CXCL8 levels were correlated with Acute Physiology and Chronic Health Evaluation (APACHE II) **(panels A, B & C)**, Murray **(panels D, E & F)** and Sequential Organ Failure Assessment (SOFA) **(panels G, H & I)** scores in patients with Tuberculosis (TB) **(panels A, D & G)**, Acute Respiratory Distress Syndrome (ARDS) **(panels B, E & H)** and the combination of both TB and ARDS **(panels C, F and I)**. Data were analysed using Pearson’s correlation of coefficient. A p value of 0.05 or less is considered significant.

There was a significant correlation between BAL CXCL-8 levels and BAL neutrophilia but this was not maintained within the miliary TB and non-miliary groups. BAL CXCL-8 levels significantly correlated with disease severity as measured by APACHE II, Murray and SOFA scores in patients with ARDS whether they had TB or not (Figure 4). In contrast, BAL CXCL-8 levels only correlated significantly with the SOFA score in patients with TB alone (Figure 5). There was no correlation between the BAL levels of IL-6, IL-1β and TNF-α and the APACHE II, Murray or SOFA scores in any of the patients groups (data not shown).

## Discussion

Here we show that the levels of CXCL8, IL-6, IL-1β and TNF-α in BAL were higher in ARDS and TB groups and in patients with combined ARDS + TB compared to control subjects. However, only the levels of BAL CXCL8 were increased in an additive manner. In addition, we demonstrated that there was a correlation between BAL CXCL-8 levels and with the severity of disease in the ARDS and the combined ARDS + TB groups. SOFA scores correlated with BAL CXCL8 levels only in the TB alone group. Differences in the Murray, APACHE II and SOFA scores were not seen with BAL IL-6, TNF-α and IL-1β levels.

TB is still one of the most important infectious diseases in the world and involved a large number of ICU patients; however the pathogenesis of ARDS induction in TB patients has not been documented. Thus, understanding the mechanism(s) involved in the pathogenesis of ARDS in TB patients is important for the development of treatments in the future. Dysregulation of the immune system plays an important role in the pathogenesis of ARDS and increased levels of CXCL8 could be an important inflammatory mediator in this disease [3]. In patients with major trauma, the levels of BAL CXCL8 were significantly higher in patients that progressed to ARDS compared to those that do not. This has been proposed to be due to acute hypoxia which can selectively up-regulate CXCL8 expression in macrophages [8]. CXCL8 is an important chemokine involved in neutrophil recruitment and has been shown to be released from inflammatory cells, such as macrophages and neutrophils, and from epithelial cells in response to inflammatory signals in ARDS [9]. As such, the increased levels of CXCL8 seen in ARDS alone could account for the increased numbers of neutrophils in the lung and lead to the damage to the respiratory system and epithelial barrier associated with disease [7]. Indeed, the distal airways of ARDS patients have greater expression of proinflammatory cytokines from airway epithelial cells and increased infiltration of macrophages and neutrophils [10]. This highlights the role of injury to the epithelium, particularly in the distal airway, in the pathophysiology of ARDS [11].

In our study we found significantly higher levels of CXCL8 and neutrophils in TB patients with ARDS than in patients with TB or ARDS alone. Thus, our current data suggests that TB could induce and maintain a local inflammatory response (increased neutrophils numbers) in the lungs. This may initiate further progression of downstream inflammatory pathways to induce ARDS with high levels of BAL CXCL8 and neutrophils. Although previous reports demonstrated increased concentrations of CXCL8, TNF-α, and IL-1β in ARDS patients compare to non-ARDS patients [12], we found that only CXCL8 increased additively in patients with ARDS induced by TB compared to patients with ARDS or TB alone. Importantly, this enhanced expression of CXCL8 correlated significantly with the Murray score. In order to determine whether ARDS in TB patients is mechanistically similar whether the disease spreads by a hematogenous or tracheobronchial route we examined the differences between subjects with milary and non-milary TB. CXCL8 expression was greater in the non-milary subjects but this difference was lost in the subjects with ARDS despite BAL neutrophil levels remaining higher. This suggests that other neutrophil chemoattractants may be involved. Further experiments will need to be performed to determine whether this difference is maintained when far greater numbers of subjects are examined. Currently, we hypothesise that although differences in the local inflammatory processes in TB occur between milary and non-milary TB, the effect of inflammation from ARDS overcomes these local differences suggesting that a similar inflammatory mechanism occurs in all subjects.

Our data suggests that CXCL8 may either be a disease-driving mediator associated with the severity of ARDS or at least a biomarker of disease severity. Our current data examining BAL inflammatory mediator levels does not clearly define the role of TNF-α and IL-1β in ARDS although it is likely that they play a role in this disease. IL-1β and TNF-*α* stimulate the synthesis and release of CXCL8 in many cell types and TB may be augmenting this pathway to induce ARDS in lungs. TB may enhance the expression of CXCL8 in the airways of ARDS patients by enhancing macrophage and neutrophil recruitment into the alveolar and bronchial wall, which would result in further release of pro-inflammatory cytokines and enhanced CXCL8 secretion from epithelial or other inflammatory cells. CXCL-8 may also further propagate the recruitment of inflammatory cells and induce damage to alveolar and bronchial wall producing ARDS. This would result in an amplification loop in these patients with TB-induced ARDS.

It is currently difficult to predict the outcome of ARDS according to clinical severity, pulmonary function tests or underlying inflammatory cytokines. In our study, we correlated the severity of ARDS with elevated CXCL8 but not with other BAL cytokines. Indeed, BAL CXCL8 levels have previously been related to the pathogenesis of ARDS, with poorer clinical outcomes and with increased disease severity and APACHE score [13]. In our study we detected a strong correlation of the Murray score with CXCL8 but not with IL-1β and TNF-α. The APACHE II score was also strongly correlated with CXCL8 levels in BAL in our study contrary to our initial hypothesis. In previous studies, the high expression of CXCL8 is associated with characteristics of severity such as low PaO_2_/FiO_2_[14].

In our study, patients with ARDS with a persistent elevation of BAL CXCL8 showed increased mortality in all ARDS patients whether induced by TB or not. There may be a causal effect since CXCL8 not only has a role in neutrophil chemotaxis but also inhibits neutrophil apoptosis [16]. Our study also confirms that BAL CXCL8 levels are associated with the severity of ARDS although there is still debate as to whether a single biomarker should be used to predict disease outcomes [15]. Together these results indicate that CXCL8 could be of predictive value for the severity and mortality of ARDS and more importantly suggests that CXCL8 inhibitors may decrease the mortality rate in these patients.

In summary, CXCL-8 concentrations are significantly higher in ARDS induced by TB and CXCL8 may have a pathogenic role in these combined diseases as they are associated with increased neutrophils. A clinical trial of CXCL8 inhibitors will determine whether CXCL8 is a direct driver of mortality and severity of these combined diseases and in ARDS alone. Further work needs to be undertaken to determine whether a combination of biomarkers and clinical predictors may be superior to clinical predictors alone for predicting mortality in ARDS.

## Abbreviations

ARDS: Acute respiratory distress syndrome; APACHE II: Acute Physiology and Chronic Health Evaluation; TB: Tuberculosis; IL: Interleukin; BAL: Bronchoalveolar lavage; ELISA: Enzyme-linked immunosorbent assay; mTB: Mycobacterium tuberculosis; SOFA: Sequential organ failure assessment; SAPS: Simplified acute physiology score.

## Competing interests

The authors declare that they have no competing interests.

## Authors’ contributions

EM wrote the original manuscript draft. SMRH and EM performed the bronchoscopies, were involved in patient recruitment and study design. PT, HJ, ZM and AK performed experimental assays and helped with patients sample collection. JG, MRM, AV, GF, PJB and IMA were involved in study design, manuscript writing and revision and data analysis. All authors read and approved the final manuscript.
